# Supplementary material for: Mexican Colorectal Cancer Research Consortium (MEX-CCRC): Etiology, Diagnosis/Prognosis, and Innovative Therapies
Source: Int J Mol Sci. 2023 Jan 20;24(3):2115. doi: 10.3390/ijms24032115 (PMC9917340; doi:10.3390/ijms24032115)
Supplement: Supplementary file 1 [file ijms-24-02115-s001.zip › ijms-2041174-supplementary.pdf]

Current members
-2022-

Unidad de Investigación en Biomedicina

Research line	Director	Manager
Cell signaling in cancer	Luis E. Arias-Romero	Olga Villamar-Cruz
Lung Diseases And Cancer Epigenomics	Federico Ávila-Moreno	Leonel Armas-López
Biochemistry and Bioenergetics	Emma B. Gutiérrez-Cirlos-Madrid	
Immunoregulation in parasitic and chronic-degenerative diseases	Luis I. Terrazas	Mónica G. Mendoza-Rodríguez
Carcinogenesis and toxicology	Yolanda I. Chirino	Norma L. Delgado-Buenrostro
Tumor immunity	Sonia Leon-Cabrera	
Immunology and host-organism interactions	Araceli Perez-Lopez	
Functional genomics of cancer	Carlos Pérez-Plasencia	Verónica García-Castillo
Experimental immunology and regulation of hepato-intestinal inflammation	José L. Reyes	
Integrative genomics and molecular biology of cancer	Felipe Vaca-Paniagua	
Innate Immunity	Miriam Rodríguez-Sosa	Imelda Juárez-Avelar

Laboratorio Nacional en Salud: Diagnóstico Molecular y Efecto Ambiental en Enfermedades Crónico-Degenerativas.

Research line	Director
Flow cytometry department	Jonadab E. Olguín
Massive sequencing department	Felipe Vaca-Paniagua

Subdirección de Investigación Básica, Instituto Nacional de Cancerología

Yessenia Sánchez-Pérez

Programa de Doctorado en Ciencias Biomédicas

Antonio Andrade-Meza

Figure S1. The current members of The Mexican Colorectal Cancer Research Consortium (MEX-CCRC).
